# Development and Performance Evaluation of Composite Modified Nano-TiO_2_ for Permeable Asphalt Mixtures: Focus on Exhaust Degradation and Dispersion Properties

**DOI:** 10.3390/ma19091777

**Published:** 2026-04-27

**Authors:** Yun Li, Shaojie Zhang, Dianliang Xi, Peilong Li, Ke Zhang, Yuefeng Zhu

**Affiliations:** 1 School of Mechanical and Electrical Engineering, Anhui Jianzhu University, Hefei 230601, China; liyunfxc@163.com; 2 School of Civil Engineering and Architecture, Anhui University of Science & Technology, Huainan 232001, China; 3 School of Highway, Chang’an University, Xi’an 710064, China; lipeilong@chd.edu.cn; 4 School of Business, Fuyang Normal University, Fuyang 236041, China; 5 College of Engineering, Computer Science, and Construction Management, California State University-Chico, Chico, CA 95929, USA

**Keywords:** permeable asphalt mixture, composite modified nano-TiO_2_, doping modification, support treatment, exhaust degradation, dispersion properties

## Abstract

Rapid urbanization has intensified challenges regarding urban waterlogging and vehicle exhaust pollution. While permeable asphalt mixtures mitigate waterlogging and nano-TiO_2_ offers photocatalytic exhaust degradation capabilities, the direct application of nano-TiO_2_ is hindered by agglomeration and low photocatalytic efficiency. This study developed a composite modified nano-TiO_2_ via metal ion doping and support treatment to enhance its performance in asphalt pavements. Specifically, nano-TiO_2_ was doped with Fe^3+^, Ag^+^, and La^3+^ via the sol–gel method, and supported on activated carbon (AC) or Al_2_O_3_. The exhaust degradation performance was evaluated using a custom-built system, while dispersion properties were assessed via fluorescence microscopy and UV-Vis spectrophotometry. Furthermore, X-ray diffraction (XRD) and Fourier-transform infrared (FTIR) spectroscopy were conducted to investigate the microstructural mechanisms underlying the doping modification and support treatment. Photocatalytic permeable asphalt mixtures were prepared by partially replacing mineral powder with the composite modified nano-TiO_2_ to validate exhaust degradation and pavement performance. The results indicated that metal doping substituted Ti^4+^ in the lattice, inducing defects and reducing crystallite size to boost photocatalytic activity. The optimal doping concentrations are determined to be 1.0% for Fe^3+^, 1.5% for Ag^+^, and 1.0% for La^3+^. Among these, Fe^3+^-doped nano-TiO_2_ at 1.0% content exhibits superior exhaust degradation, achieving 46.7% efficiency for hydrocarbons (HC) and 33.5% for nitrogen oxides (NO). Regarding dispersion, while AC performs better at low support content, Al_2_O_3_ at 40% content provides superior dispersion properties by increasing active sites and surface hydroxyl groups. For photocatalytic permeable asphalt mixtures, replacing 40–50% of mineral filler with the composite modifier is recommended. The optimized mixture demonstrates superior exhaust degradation performance while maintaining the required high-temperature stability, low-temperature cracking resistance, water stability, and fatigue life. Specifically, compared to the control group, these indicators for the mixture with 50% of the mineral filler replaced by the composite modifier increases by 7.0%, 12.5%, 13.4%, and 22.9%, respectively. This study presents a viable technical solution for developing multifunctional asphalt mixtures with photocatalytic functionality as the core innovation and mechanical performance as the application baseline.

## 1. Introduction

In recent years, urban waterlogging during rainfall has become a persistent problem in many large and medium-sized cities [1]. Meanwhile, with social development, frequent and large-scale haze events have occurred in numerous cities. Excessively high concentrations of PM2.5 in the atmosphere are the primary cause of haze, and vehicle exhaust emitted during fuel combustion represents the major mobile source of PM2.5. With the continuous growth in number of vehicles in traffic, vehicle exhaust has become the dominant contributor to urban PM2.5 pollution, ranking first among all emission sources. Vehicle exhaust poses a severe threat to the atmospheric environment and human health, and has become one of the main causes of urban haze [2]. Urban roads cover extensive areas, serving as a critical component of urban infrastructure. The dual challenges of frequent waterlogging and worsening vehicle exhaust pollution have imposed stringent requirements on the ecological and environmental performance of urban roads. Therefore, it is particularly important for urban pavements to possess both rapid drainage and exhaust purification functions [3].

Nano-TiO_2_ is widely employed as air-purification material owing to its excellent photocatalytic activity, chemical stability, and recyclability [4,5,6]. Based on the photocatalytic effect of nano-TiO_2_, the purification of nitrogen oxides (NOₓ) and hydrocarbons (HC) in vehicle exhaust can be effectively achieved. To date, numerous scientists have conducted extensive research on vehicle exhaust purification using nano-TiO_2_. Khiavi et al. [7] investigated the influence of nano-TiO_2_ as an additive on the pollutant absorption efficiency of micro-surfacing mixtures. The absorption rate of NOₓ increased with the nano-TiO_2_ content, reaching 40% at a dosage of 11% by weight of asphalt. Fang et al. [8] proposed a strategy of incorporating nano-TiO_2_ into pavement marking materials to reduce NOₓ from vehicle exhaust. Guo et al. [9] added photocatalytic mixed-crystal nano-TiO_2_ particles into concrete via internal doping and spraying, revealing that illumination conditions affect the photocatalytic degradation efficiency of nano-TiO_2_-modified concrete. Xu et al. [10,11] combined nano-TiO_2_ with fog seal technology, investigated the influences of material and environmental factors on three-dimensionally ordered macroporous (3DOM) TiO_2_ fog seals, and proposed measures to improve the exhaust degradation efficiency of 3DOM TiO_2_ materials. Liang et al. [12] prepared five rubberized open-graded (ROG) asphalt mixtures with different TiO_2_ contents, observing that TiO_2_ exhibited significant absorption effects on CO, HC, and NO, and recommended an optimal TiO_2_ dosage of 0.4%.

Doping is defined as the intentional introduction of impurity elements into a semi-conductor lattice to alter its physical and chemical properties [13]. Recently, Hu et al. [14,15] developed green and sustainable photocatalytic pavement materials for vehicle exhaust purification, and confirmed that Fe^3+^ ion doping enhances the catalytic efficiency of TiO_2_ under visible light irradiation. Wu et al. [16] prepared TiO_2_ photocatalysts with different Ag^+^ contents, demonstrating that Ag^+^ deposition on TiO_2_ narrows the band gap and enhances visible-light absorption. The 5% Ag^+^ modified TiO_2_ sample exhibited superior photocatalytic activity. Qian et al. [17] synthesized three composite photocatalytic coating nanomaterials and evaluated their NO degradation efficiency, with Fe_2_O_3_ modified TiO_2_ showing the best performance. Alcantara et al. [18] verified the potential of chitosan–TiO_2_ (CS–TiO_2_) functionalized asphalt mixtures in reducing high NO_2_ concentrations and improving durability. Zhang et al. [19] incorporated nano-TiO_2_ into low-carbon concrete, finding that it effectively modified the mechanical properties, pore structure, and photocatalytic performance of hardened concrete, thus enhancing self-cleaning efficiency. Xu et al. [20] prepared BOCN–TiO_2_ with a large specific surface area via composite modification, reporting that its degradation capacity for NO and HC is more than twice that of traditional g-C_3_N_4_. Shan et al. [21] synthesized Bi-doped TiO_2_ nanoparticles via sol–gel and hydrothermal methods, achieving a NO degradation efficiency of 77.6% under visible light. Moreover, Bukichev et al. [22] revealed that nano-TiO_2_ tends to agglomerate when introduced into polymer matrices, and the dispersion uniformity affects the mechanical properties of composites. Lei et al. [23] found that activated carbon (AC) support improves the dispersion of nano-TiO_2_, and the Fe^3+^-TiO_2_-AC composite catalyst exhibits better degradation of CO, NO, and HC than pure nano-TiO_2_.

Porous asphalt pavements enable rapid drainage of urban roads through the porous structure, which efficiently drains surface water in a timely manner. The incorporation of photocatalytic materials into porous asphalt mixtures allows sufficient contact between vehicle exhaust and catalytic materials due to the high porosity of the mixture, thereby effectively improving the photocatalytic degradation efficiency of gaseous pollutants [24]. Yan et al. [3] employed porous asphalt mixtures as supports for photocatalytic pavements and systematically investigated the effects of void ratio, pavement thickness, and type on exhaust degradation performance, providing valuable guidelines for structural design. However, despite these advancements, current applications of nano-TiO_2_ for exhaust degradation mainly involve direct mixing into asphalt mixtures or surface coating on pavements, often neglecting the unique challenges of the asphalt binder environment. The activation of nano-TiO_2_ requires ultraviolet light with a wavelength shorter than 387.5 nm, and the catalytic activity cannot be fully exerted in the visible light region [25]. Meanwhile, the high recombination rate of photoinduced electron–hole pairs in nano-TiO_2_ also limits the catalytic efficiency [26]. Most importantly, nano-TiO_2_ is difficult to disperse uniformly due to the small particle size and often agglomerates in asphalt mixtures, which reduces the effective active surface area within the porous structure [27]. Existing studies often address these issues separately, such as using doping modification to improve activity or using support to enhance dispersion, and lack a systematic composite strategy tailored for permeable asphalt mixtures. Therefore, the development of environment-friendly asphalt mixtures with both exhaust degradation and permeable functions requires systematic research on the synergistic effects of catalytic efficiency and dispersion behavior of nano-TiO_2_, as well as validation of the pavement performance of nano-TiO_2_-modified asphalt mixtures.

To bridge this gap, the novelty of this study lies in the development of a composite modified nano-TiO_2_ via a synergistic strategy of metal ion doping and support treatment. The primary objective is to effectively enhance the photocatalytic exhaust degradation performance of permeable asphalt mixtures while quantitatively evaluating the dispersion properties. Meanwhile, the pavement performance (including high-temperature stability, low-temperature cracking resistance, and water stability) was evaluated as a critical constraint to ensure engineering feasibility. Therefore, the development of environment-friendly asphalt mixtures with both exhaust degradation and dispersion optimization is the central focus, with mechanical stability serving as the necessary prerequisite for application. This approach aims to provide a viable technical solution for developing multifunctional asphalt mixtures that balance environmental functionality with engineering reliability.

## 2. Materials and Methods

### 2.1. Raw Materials

#### 2.1.1. Development of Composite Modified Nano-TiO_2_

The laboratory synthesis of composite modified nano-TiO_2_ utilized the following raw materials: butyl titanate, iron (III) nitrate nonahydrate, lanthanum nitrate hexahydrate, absolute ethanol, concentrated hydrochloric acid, silver nitrate, concentrated nitric acid, and glacial acetic acid, as detailed in Table 1. Activated carbon (AC) and aluminum oxide (Al_2_O_3_) supports were purchased from Tianjin Dengfeng Chemical Reagent Factory (Tianjin, China) and were of analytical reagent (AR) grade. According to the manufacturer’s specifications, the AC typically exhibits a specific surface area of 800–1200 m^2^/g, pore volume of 0.4–0.8 cm^3^/g, and a particle size of 100–200 mesh. The Al_2_O_3_ support typically has a specific surface area of 150–250 m^2^/g, pore volume of 0.35–0.6 cm^3^/g, and a particle size of 200–300 mesh.

**Figure 1:**
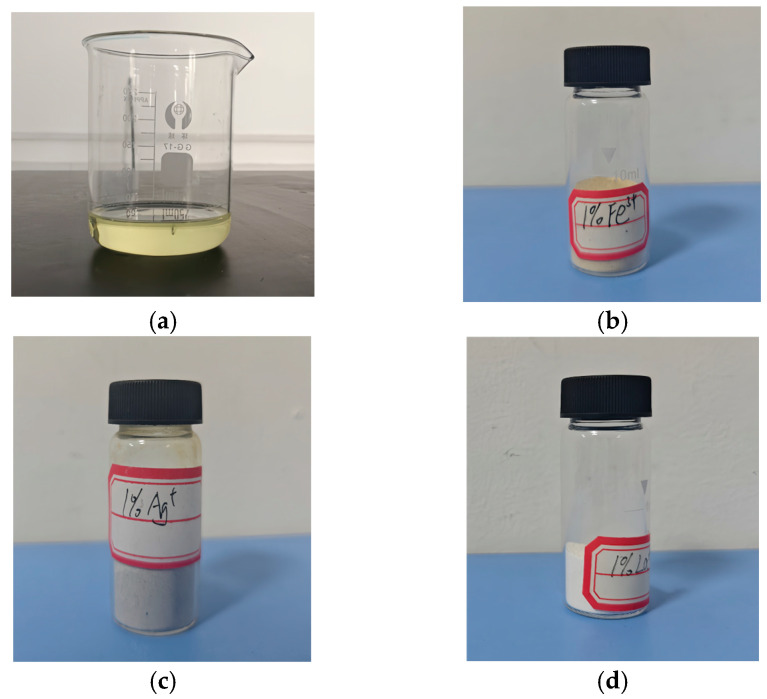
Metal ion-doped modified nano-TiO_2_. (**a**) Solution A; (**b**) 1.0% Fe^3+^; (**c**) 1.0% Ag^+^; (**d**) 1.0% La^3+^.

The metal ion-doped nano-TiO_2_ was fabricated via a sol–gel process involving the following steps:

First, Solution A was prepared by mixing absolute ethanol (25 mL) and glacial acetic acid (5 mL) in a beaker, followed by the addition of butyl titanate (15 mL). The beaker was sealed with plastic wrap during magnetic stirring for 30 min at room temperature to inhibit premature hydrolysis and prevent splashing, resulting in a homogeneous, pale yellow solution (Figure 1a).

Second, Solution B was prepared by mixing absolute ethanol (20 mL), distilled water (10 mL), and glacial acetic acid (5 mL). Metal precursors (iron(III) nitrate nonahydrate, silver nitrate, and lanthanum nitrate hexahydrate) were added to achieve metal ion-to-Ti molar ratios of 0.5%, 1%, 1.5%, and 2%. The pH was adjusted to 2–3 using concentrated hydrochloric acid. For gelation, Solution B was added dropwise to Solution A via a dropping funnel at a rate of 1–2 drops/s. The resulting sol was aged at room temperature for 48–72 h to form a gel [21].

Finally, the gel was dried at 80 °C for 12 h in a blast oven to produce a xerogel [28]. The xerogel was ground and calcined at 500 °C for 2 h in a muffle furnace to obtain the metal ion-doped nano-TiO_2_ (Figure 1b–d).

Subsequently, support treatment was performed on 1.0% Fe^3+^ doped nano-TiO_2_ using activated carbon (AC) and aluminum oxide (Al_2_O_3_) as follows:

AC and Al_2_O_3_ were dispersed in a mixture of absolute ethanol (20 mL), distilled water (10 mL), and glacial acetic acid (5 mL) using ultrasonic agitation for 30 min to form solution D. The support contents (AC/Al_2_O_3_ to Ti) were set at 20%, 30%, 40%, and 50%, with the pH adjusted to 2–3. Solution D was added dropwise to the Fe^3+^-doped sol (Solution C) at 1–2 drops/s. The mixture was aged for 48–72 h to form a composite gel. The final composite was dried at 80 °C for 12 h, ground into powder, and calcined at 500 °C for 2 h.

**Figure 2:**
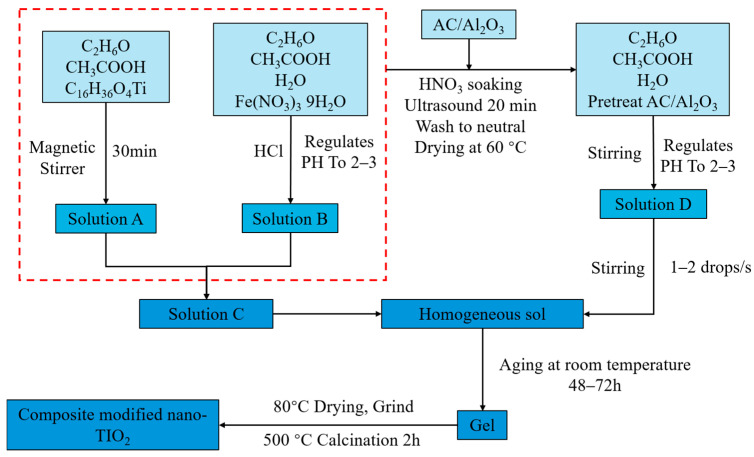
Preparation flow of composite modified nano-TiO_2_.

The overall preparation scheme is illustrated in Figure 2.

#### 2.1.2. Preparation of Photocatalytic Permeable Asphalt Mixture

**Figure 3:**
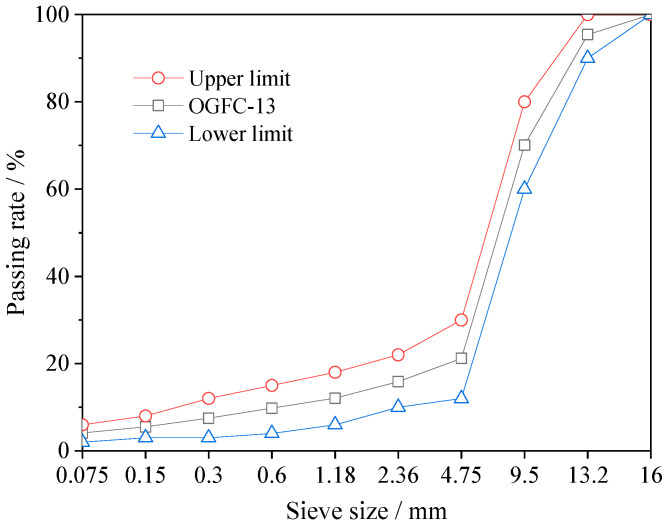
Aggregate gradation of photocatalytic permeable asphalt mixture.

The gradation was selected as an open-graded structure to ensure interconnected voids. Specifically, the photocatalytic permeable asphalt mixture was designed with an OGFC-13 aggregate gradation, and the gradation curve is shown in Figure 3. Composite modified nano-TiO_2_ was used to replace the mineral filler at mass ratios of 0%, 30%, 40%, 50%, and 60%. The binder used was SBS modified asphalt, satisfying the specification requirements.

To determine the optimal asphalt content, a series of mixtures with asphalt–aggregate ratios ranging from 4.0% to 6.0% (in 0.5% increments) were prepared. The allowable range of asphalt content was established through drainage and Cantabro tests to prevent bleeding and raveling, respectively [29]. Specifically, the specimens of permeable asphalt mixture for Cantabro tests were prepared using the Marshall compaction method. The mixtures were compacted with 50 blows on each side. Based on these criteria, the optimal asphalt–aggregate ratio was determined to be 4.75%. At this dosage, the mixture achieved an air void content of 21.4% and a permeability coefficient of 1264 mL/min, which complies with the required specifications for permeable asphalt pavement [30]. The mixture gradation was uniformed by the dual targets of water permeability to mitigate waterlogging and gas diffusion to ensure exhaust gases can fully contact the nano-TiO_2_ [31].

### 2.2. Test Methods

#### 2.2.1. Exhaust Degradation Effect of Composite Modified Nano-TiO_2_

**Figure 4:**
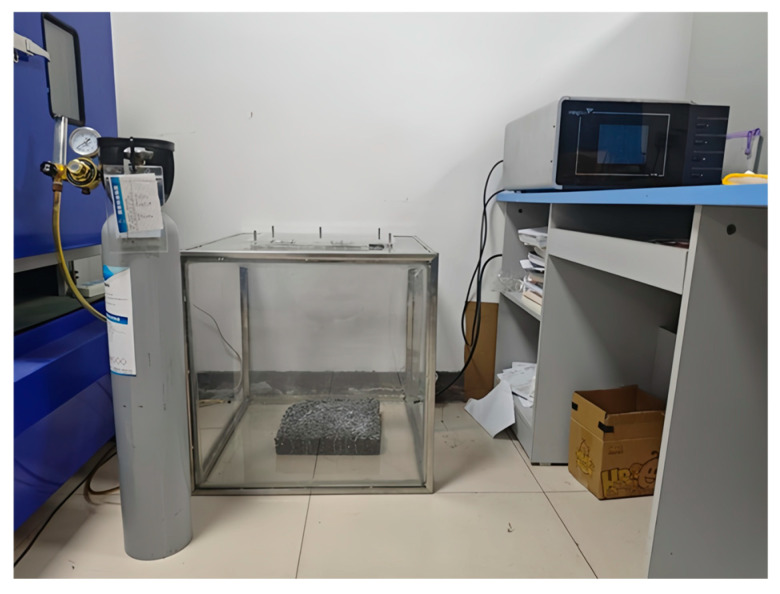
Test on the degradation effect of vehicle exhaust.

To evaluate the degradation performance of the developed composite-modified nano-TiO_2_ on vehicle exhaust, a specialized testing system suitable for asphalt pavement was designed in collaboration with Fuyang Xiaguang Motor Vehicle Testing Co., Ltd. (Fuyang, China). As illustrated in Figure 4, the system primarily comprises three components: an exhaust gas cylinder, a reaction chamber, and an exhaust gas analyzer, which is consistent with previous studies [3]. The photocatalytic reaction was driven by a 100 W simulated sunlight lamp providing full-spectrum irradiation to activate the photocatalyst. The light intensity was 27.8 mW/cm^2^, and the lamp was placed 55 cm vertically above the samples to ensure uniform illumination. The exhaust gas cylinder is a customized standard tank containing pollutant gases, such as hydrocarbons (HC) and nitrogen monoxide (NO), enabling precise control of the initial gas concentration. The exhaust gas analyzer with the model of MQW-50 made by Zhejiang Mingquan Technology Co., Ltd. (Hangzhou, China) enables real-time monitoring of pollutant concentration changes.

For the coating preparation, 0.5 g of composite modified nano-TiO_2_ powder was dispersed in 50 g of distilled water at a ratio of 1:100. A high-speed shear mixer homogenized the mixture at 1500 r/min for 30 min to prepare a water-based modified TiO_2_ coating solution. This solution was uniformly applied to a standard rutting plate (30 cm × 30 cm× 5 cm) and allowed to dry at room conditions prior to testing. Additionally, the plate specimens of the photocatalytic permeable asphalt mixture coated with unmodified nano-TiO_2_ solution were also prepared for exhaust degradation test. The number of parallel samples for exhaust degradation test was 3 [32].

The photocatalytic degradation tests were carried out at a controlled temperature of 25 °C, with relative humidity maintained under consistent ambient laboratory conditions. The photocatalytic degradation rate was defined as the reduction in exhaust concentration per unit reaction time. The degradation rate was calculated using Equation (1).

(1) $$\eta =\frac{(N_{t1}-N_{t2})-N_{W}}{\Delta t}$$

where *η* is the degradation rate, ppm/min; *N_t_*_1_ and *N_t_*_2_ represent the gas concentrations (ppm) of the photocatalytic sample group at times t_1_ and t_2_, respectively, ppm; and Δt refers to the reaction interval, with t2 − t1 = 20 min. The term (*N_t_*_1_ − *N_t_*_2_) represents the total concentration reduction observed in the experimental group, ppm.

To isolate the photocatalytic effect from physical interference, a blank correction value *N_W_* was introduced, defined as:

(2) $$N_{w}=N_{t1}-N_{t2}$$

where *N_t_*_1_ and *N_t_*_2_ are the gas concentrations of the blank control group without nano-TiO_2_, ppm. The value N_W_ represents the concentration loss due to non-photocatalytic factors such as physical adsorption, or natural decay. Therefore, subtracting *N_W_* from the sample’s total concentration difference ensures that the calculated degradation rate reflects only the net photocatalytic activity, excluding background interference.

Degradation efficiency served as the primary evaluation index for the vehicle exhaust degradation performance. It was defined as the percentage of vehicle exhaust catalytically degraded by the composite modified nano-TiO_2_ relative to the initial concentration over the period from 0 to 120 min, as shown in Equation (3) [15].

(3) $$D=\frac{N_{0}-N_{120}-{N_{W}}^{\prime }}{N_{0}}\times 100\%$$

where *D* is the catalytic degradation efficiency, %; *N*_0_ is the initial concentration, ppm; *N*_120_ is the concentration at 120 min, ppm; *N_W_^′^* is the correction value from 0 to 120 min, ppm.

#### 2.2.2. Dispersion Characteristics of Composite Modified Nano-TiO_2_

(1) Fluorescence microscopy observation.

**Figure 5:**
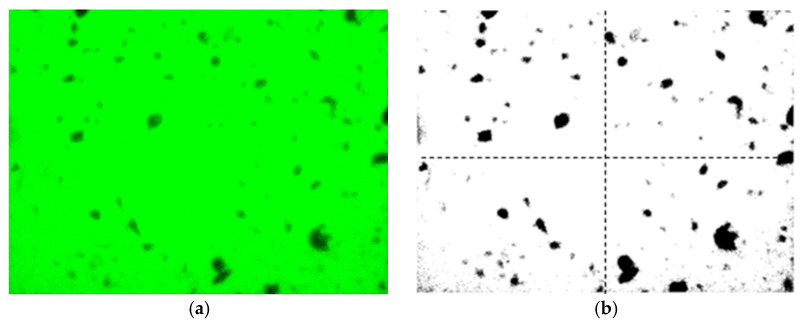
Dispersion effect diagram. (**a**) Fluorescence micrograph; (**b**) Binary image.

To evaluate the dispersion of composite modified nano-TiO_2_ within the asphalt binder following support treatment with AC and Al_2_O_3_, a fluorescence tracing method was employed [33]. The composite modified nano-TiO_2_ was homogenized with a fluorescent dye (C_20_H_12_O_5_) at a mass ratio of 100:5. Subsequently, this mixture was pre-dispersed with the asphalt binder at a ratio of 4:100 using ultrasonic vibration for 30 min followed by high-speed shear mixing at 160 °C for 15 min. Micrographs of the asphalt samples were captured using a fluorescence microscope. Image analysis was conducted using MATLAB R2024a software. The acquired images were converted into 8-bit grayscale format. Subsequently, the images were converted into binary format using Otsu’s thresholding algorithm to automatically determine the optimal threshold separating the nano-TiO_2_ modifier from the asphalt background. This automated approach minimizes manual bias and ensures consistency in pixel quantification [34]. The binary images were then segmented into four equal regions, as illustrated in Figure 5.

The pixel counts for the black areas and white areas in each region were quantified. The area ratio of the black phase in each region and the range of these area ratios were calculated using Equations (4) and (5), respectively.

(4) $$S_{i}=\frac{M_{i}}{M_{i}+N_{i}}$$

(5) $$S=\left|S_{\max}-S_{\min}\right|$$

where S_i_ is the black area ratio in the i-th region; *M_i_* and *N_i_* are the pixel counts of the black and white areas in the i-th region, respectively; S denotes the range of the black area ratio; *S_max_* and *S_min_* represent the maximum and minimum black area ratios among the four regions, respectively.

(2) UV-Vis spectrophotometer test.

Furthermore, UV-Vis spectrophotometry was utilized to test the dispersion uniformity of the composite-modified nano-TiO_2_. Specifically, 20 mL of absolute ethanol was placed in a beaker, to which modified nano-TiO_2_ samples (prepared with varying AC and Al_2_O_3_ support contents) were added. The mixtures underwent ultrasonication for 30 min to prepare suspensions. After allowing the suspensions to settle for 12 h, the sedimentation behavior was visually observed. This settling duration was selected based on preliminary time-dependent experiments, where significant differences in sedimentation behavior between samples became distinct and stabilized within this period [35]. Subsequently, the absorbance of the upper liquid was measured using a UV-Vis spectrophotometer. The instrument model is UV-1950, manufactured by Beijing Puxi General Instrument Co., Ltd. (Beijing, China). The number of parallel samples for UV-Vis spectrophotometer test was 3. Higher absorbance values in the supernatant indicate better dispersion uniformity and less sedimentation of the nanoparticles [36].

#### 2.2.3. Microscopic Properties of Composite Modified Nano-TiO_2_

(1) XRD test.

XRD is a fundamental technique used to characterize the crystal structure and phase composition of materials. Based on Bragg’s law, X-rays interacting with atomic lattices produce diffraction patterns via constructive interference, facilitating the determination of interplanar spacing and material identification. XRD analysis was performed on the nano-TiO_2_ samples doped with metal ions (Fe^3+^, Ag^+^, La^3+^) and loaded with supports (AC and Al_2_O_3_) to verify their crystalline phases.

Lattice parameters serve as sensitive indicators of subtle structural variations induced by metal ion doping. To further investigate the influence of metal ion type and doping content on the crystallite size, the values were calculated using the Scherrer equation (Equation (6)) [37].

(6) $$D=\frac{K\lambda }{B\cos\theta }$$

Specifically, the full width at half maximum (FWHM) of the most intense anatase (101) diffraction peak located at 25.6° was selected for this calculation to ensure consistency across all doped samples. In the equation, *D* is the crystallite size, nm; *K* is the Scherrer constant (typically 0.89); *λ* is the X-ray wavelength, nm; *B* is the FWHM of the diffraction peak, rad; and *θ* is the Bragg diffraction angle, °.

(2) FTIR test.

FTIR was employed to investigate the molecular structure and functional group composition of the samples. This technique relies on the absorption of infrared radiation by chemical bonds, which undergo vibrational transitions at specific frequencies [38]. Characteristic absorption peaks corresponding to specific bonds of C-H, O-H and C=O were analyzed to identify chemical interactions. FTIR tests were conducted on the modified nano-TiO_2_, including those doped with Fe^3+^, Ag^+^, and La^3+^, as well as those supported with AC and Al_2_O_3_.

#### 2.2.4. Pavement Performance of Photocatalytic Permeable Asphalt Mixture

Photocatalytic permeable asphalt mixture specimens were prepared by substituting mineral filler with composite modified nano-TiO_2_ at replacement ratios of 30%, 40%, 50%, and 60% by weight. The composite modified nano-TiO_2_ was mixed with aggregate and SBS modified asphalt according to the standard mixing process of permeable asphalt mixture to ensure uniform dispersion [3]. The control group consisted of unmodified permeable asphalt mixtures were also prepared. To comprehensively evaluate the performance of the photocatalytic permeable asphalt mixture, a series of mechanical tests were conducted, including the dynamic water scouring Marshall test, low-temperature splitting test, rutting test, and four-point bending fatigue test. The number of parallel samples for pavement performance test was 3. The average value of three parallel samples was taken as the representative value. These tests assess water stability, low-temperature crack resistance, high-temperature stability, and fatigue resistance, respectively.

The detailed test parameters and evaluation indexes were as follows: An SYD-0777 hydrothermal sensitivity tester made by Shanghai Changji Geological Instrument Co., Ltd. (Shanghai, China) was employed to subject Marshall specimens to water scouring at 30 °C and 200 kPa for 3500 cycles. The scouring residual stability ratio was adopted as the evaluation index for water stability. The low-temperature splitting test was conducted at −10 °C with a loading rate of 50 mm/min. The splitting strength served as the performance indicator for low-temperature crack resistance. The rutting test was performed at 60 °C under a wheel pressure of 0.7 MPa. Dynamic stability was calculated to evaluate high-temperature stability. The fatigue test was carried out at 15 °C with a loading frequency of 10 Hz. Fatigue life was recorded as the index for fatigue resistance [31].

## 3. Results and Discussion

### 3.1. Evaluation of Exhaust Degradation Effect by Modified Nano-TiO_2_

#### 3.1.1. HC Degradation Effect Analysis

**Figure 6:**
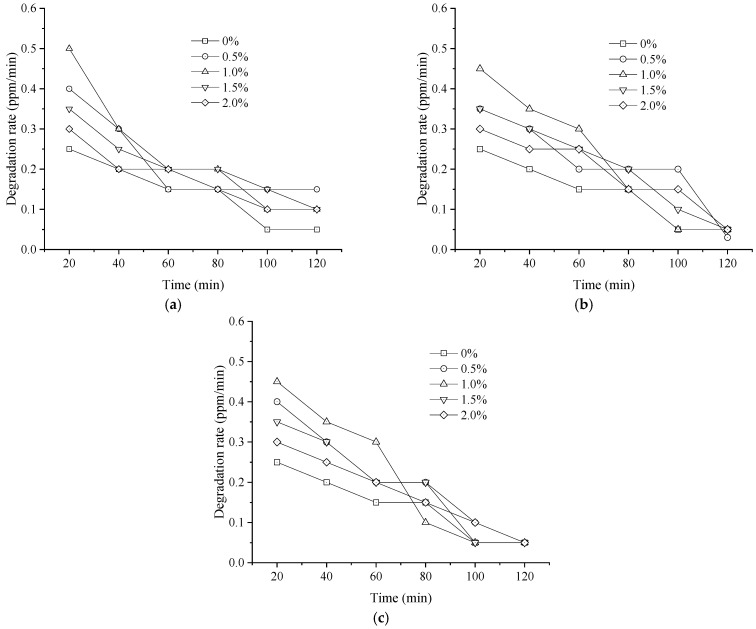
Average HC degradation rate over 20 min intervals for metal ion-doped modified nano-TiO_2_. (**a**) Fe^3+^; (**b**) Ag^+^; (**c**) La^3+^.

**Figure 7:**
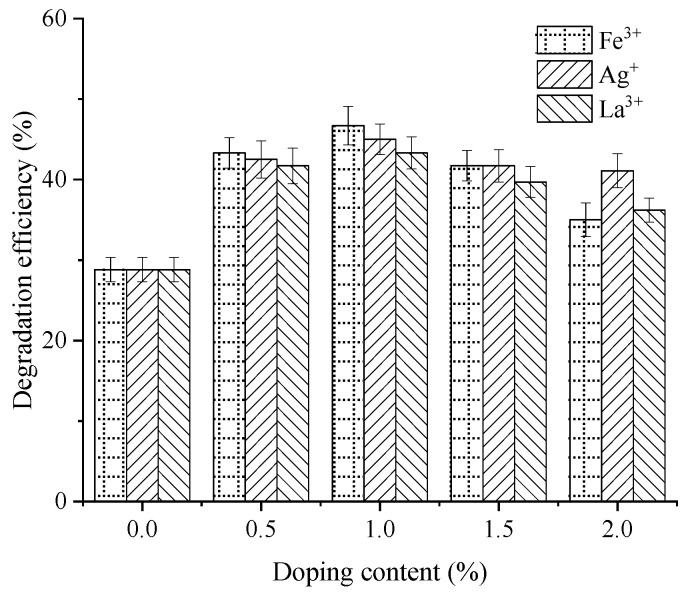
HC degradation efficiency of metal ion-doped modified nano-TiO_2_.

Figure 6 and Figure 7 illustrated the effects of metal ion type and doping content on the degradation rate and efficiency of HC over a 120 min period, including a control group containing unmodified nano-TiO_2_.

As shown in Figure 6 and Figure 7, the HC degradation rate exhibits a gradual decline over time across all Fe^3+^, Ag^+^, and La^3+^ doping levels. The gradual decline in degradation rate over time is not due to deactivation of the modified nano-TiO_2_ but reflects the photocatalytic kinetics, where the reaction rate decreases as the pollutant concentration is consumed [39]. The unmodified nano-TiO_2_ exhibited a slow reduction in degradation rate, whereas modification with metal ions results in a notably higher initial rate. The degradation process could be divided into three stages based on the rate profile: a rapid degradation (0–60 min), a slow degradation (60–100 min), and a stable stage (100–120 min). The degradation process transitions from an initial rapid stage driven by high concentration gradients and abundant active sites, to a slow stage limited by decreased collision frequency and intermediate competition, and finally to a stable stage reflecting adsorption–desorption equilibrium at low concentrations [3]. Taking the initial 20 min interval as an example, the degradation rate for unmodified nano-TiO_2_ was 0.25 ppm/min. These rates were calculated using Equation (1) over a fixed time interval (Δt = 20 min). Metal ion doping markedly enhances this rate. Specifically, at doping contents of 0.5%, 1.0%, 1.5%, and 2.0%, the HC degradation rates for Fe^3+^-doped nano-TiO_2_ were 0.40, 0.50, 0.35, and 0.30 ppm/min, respectively. For Ag^+^-doped nano-TiO_2_, the rates were 0.35, 0.45, 0.35, and 0.30 ppm/min. For La^3+^-doped nano-TiO_2_, they were 0.40, 0.45, 0.35, and 0.30 ppm/min. Notably, the 1.0% Fe^3+^-doped sample exhibited the highest degradation rate (0.50 ppm/min) in this interval, outperforming the Ag^+^ and La^3+^ samples at the same concentration. The influence of metal ion type and content on HC degradation rate in other time intervals was consistent with the trends observed at 0–20 min.

Meanwhile, substantial differences in the cumulative HC degradation efficiency were evident among the various composite modifiers after 120 min. The unmodified nano-TiO_2_ achieved an efficiency of 28.8%. For Fe^3+^-doped samples (0.5%, 1.0%, 1.5%, and 2.0%), efficiencies reached 43.3%, 46.7%, 41.7%, and 35.0%, corresponding to increases of 50.4%, 62.0%, 44.6%, and 21.5% over the unmodified control, respectively. For Ag^+^-doped samples, efficiencies were 42.5%, 45.0%, 41.7%, and 41.1%, representing improvements of 47.5%, 56.2%, 44.6%, and 42.5%. For La^3+^-doped samples, efficiencies were 41.7%, 43.3%, 39.7%, and 36.2%, reflecting increases of 44.6%, 50.3%, 37.6%, and 25.7%. Evidently, the optimal HC degradation efficiency was achieved at a doping content of 1.0% for Fe^3+^, Ag^+^, and La^3+^ during material preparation. At this concentration, the HC degradation efficiencies for Fe^3+^, Ag^+^, and La^3+^-modified nano-TiO_2_ reached 46.7%, 45.0%, and 43.3%, respectively. Moreover, to contextualize these findings, the results were benchmarked with recent studies on nano-modified pavements. Compared to single-doped nano-TiO_2_ studies in semi-flexible pavements [14] or cement mortar [16], the composite modified nano-TiO_2_ in this study achieved higher HC degradation efficiency, attributed to the synergistic effects of metal ion doping and support treatment [23].

#### 3.1.2. NO Degradation Effect Analysis

**Figure 8:**
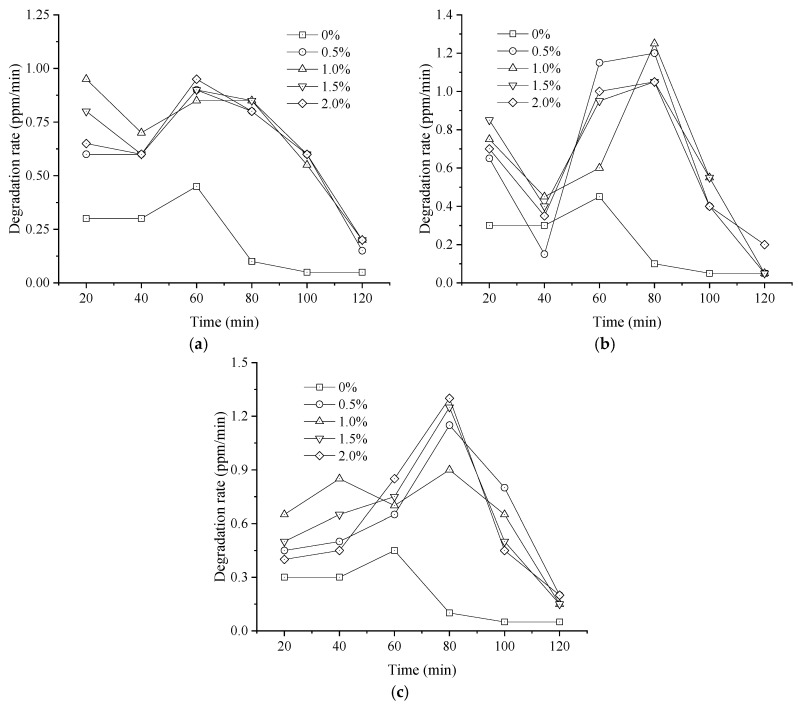
Average NO degradation rate over 20 min intervals for metal ion-doped modified nano-TiO_2_. (**a**) Fe^3+^; (**b**) Ag^+^; (**c**) La^3+^.

**Figure 9:**
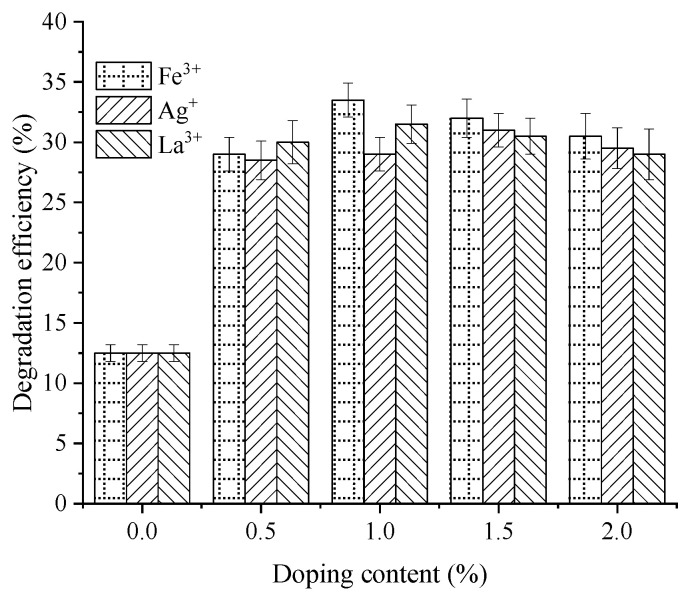
NO degradation efficiency of metal ion-doped modified nano-TiO_2_.

The effects of modified nano-TiO_2_ with varying Fe^3+^, Ag^+^, and La^3+^ contents on NO degradation rate and efficiency within 120 min are illustrated in Figure 8 and Figure 9. The control group for this test consisted of asphalt mixture specimens containing unmodified nano-TiO_2_.

As shown in Figure 8 and Figure 9, the NO degradation rate of modified nano-TiO_2_ fluctuated over time under different doping contents, with relatively high rates observed during the 0–20 min and 60–80 min intervals. These rates were calculated based on the concentration difference over each 20 min interval. In the initial 20 min interval, the NO degradation rate of the control group was 0.30 ppm/min. Compared with the control group, at metal ion contents of 0.5%, 1.0%, 1.5%, and 2.0%, the NO degradation rates for Fe^3+^-doped nano-TiO_2_ increased by 100.0%, 216.7%, 166.7%, and 116.7%, respectively. For Ag^+^-doped nano-TiO_2_, the degradation rates increased by 116.7%, 150.0%, 183.3%, and 133.3%. For La^3+^-doped nano-TiO_2_, the rates increased by 50.0%, 116.7%, 66.7%, and 33.3%. The maximum NO degradation rates were observed at doping contents of 1.0% for Fe^3+^, 1.5% for Ag^+^, and 1.0% for La^3+^. Furthermore, the NO degradation efficiency of Fe^3+^-doped nano-TiO_2_ was generally higher than that of the Ag^+^ and La^3+^ variants.

Moreover, regarding total efficiency over 120 min, the unmodified nano-TiO_2_ reached 12.5%. For Fe^3+^-doped samples (0.5%, 1.0%, 1.5%, and 2.0%), NO degradation efficiencies were 29.0%, 33.5%, 32.0%, and 30.5%. These values represent increases of 132.0%, 168.0%, 156.0%, and 144.0% compared with the unmodified group. For Ag^+^-doped samples, the efficiency improvements relative to the control were 128.0%, 132.0%, 148.0%, and 136.0% for doping contents of 0.5%, 1.0%, 1.5%, and 2.0%, respectively. For La^3+^-doped samples, efficiencies increased by 140.0%, 152.0%, 144.0%, and 132.0%. Consequently, the peak NO degradation efficiencies were achieved at Fe^3+^, Ag^+^, and La^3+^ contents of 1.0%, 1.5%, and 1.0%, respectively, reaching 33.5%, 31.0%, and 31.5%.

### 3.2. Evaluation of Dispersion Properties of Composite Modified Nano-TiO_2_

#### 3.2.1. Quantitative Analysis of Dispersion Uniformity

**Figure 10:**
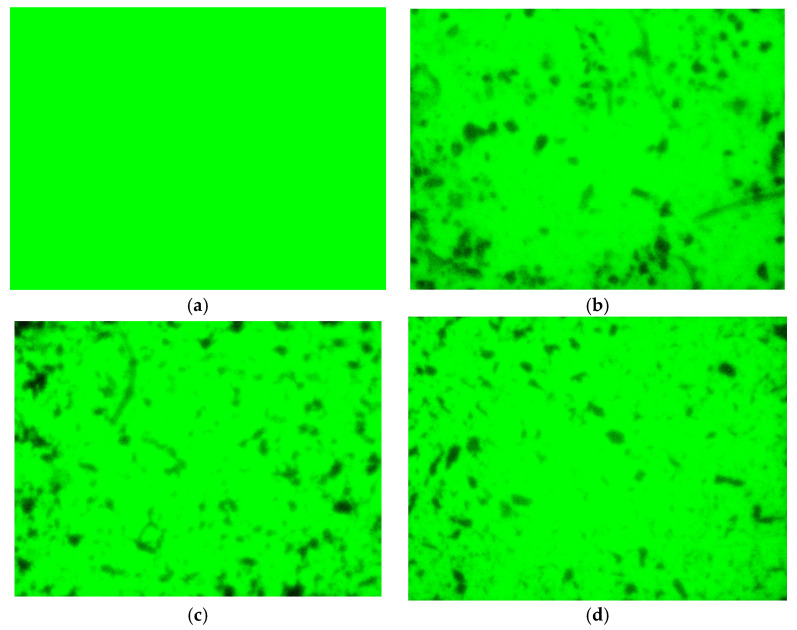
Dispersion effect of composite modified nano-TiO_2_ in asphalt. (**a**) Asphalt with fluorescent dye; (**b**) TiO_2_ without support; (**c**) 40% AC; (**d**) 40% Al_2_O_3_.

AC and Al_2_O_3_ were employed as supports for Fe^3+^-doped modified nano-TiO_2_ (1.0% doping content). The dispersion of the composite modified nano-TiO_2_ in asphalt under varying AC and Al_2_O_3_ contents is illustrated in Figure 10.

As shown in Figure 10, the asphalt doped solely with the fluorescent dye exhibited a homogeneous green background. This indicated that the fluorescent dye added to the asphalt does not interfere with the subsequently loaded nano-TiO_2_. Compared with the modified nano-TiO_2_ without support, AC and Al_2_O_3_ supports improved the dispersion of modified nano-TiO_2_ within the asphalt binder and effectively reduced agglomeration. This enhancement increased the contact area between the modified nano-TiO_2_ and exhaust gases, thereby boosting exhaust gas degradation efficiency. However, the benefit of the support treatment on dispersion diminished as the support content increased beyond a certain threshold.

**Figure 11:**
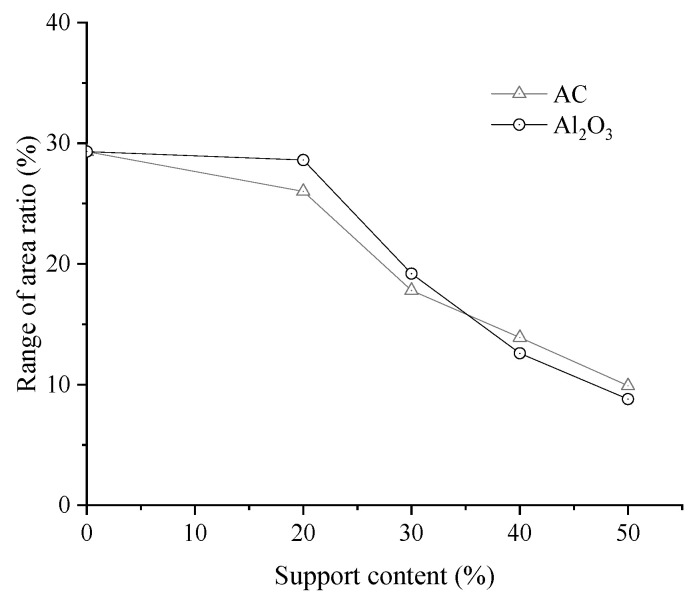
Range of area ratios of composite modified nano-TiO_2_.

To quantitatively analyze the effect of AC and Al_2_O_3_ supports on dispersion, the asphalt images were processed using MATLAB. The range of area ratios of the black regions under different support conditions is presented in Figure 11.

As illustrated in Figure 11, both the type of supports and the support content influenced the dispersion of modified nano-TiO_2_ in asphalt. With increasing support content, the dispersion uniformity of the composite initially improved and subsequently stabilized. The range of area ratios for unsupported nano-TiO_2_ was 29.3%. At support contents of 20%, 30%, 40%, and 50%, the ranges of area ratios for AC-supported nano-TiO_2_ decreased by 11.3%, 39.2%, 52.6%, and 66.2%, respectively. For Al_2_O_3_-supported nano-TiO_2_, the corresponding decreases were 2.4%, 34.5%, 57.0%, and 70.0%. At lower support contents (20% and 30%), the ranges of area ratios of AC-supported samples were lower than those of the Al_2_O_3_-supported samples, indicating more uniform dispersion. However, after the support content exceeded 40%, the Al_2_O_3_-supported samples exhibited a smaller range of area ratios, reflecting superior compatibility and bonding strength between Al_2_O_3_ and the modified nano-TiO_2_. This ensured uniform dispersion, minimized agglomeration, and maximizes the contact area with asphalt, thereby enhancing exhaust gas degradation performance.

#### 3.2.2. Characterization and Verification of Dispersion Uniformity

**Figure 12:**
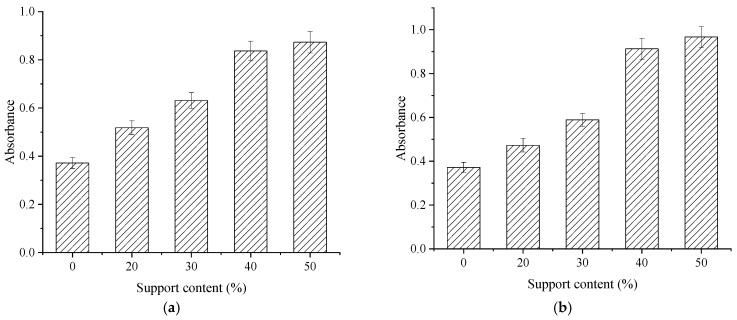
Absorbance of composite modified nano-TiO_2_ suspensions. (**a**) AC; (**b**) Al_2_O_3_.

To further verify the dispersion uniformity of the composite modified nano-TiO_2_, UV–Vis spectrophotometry was conducted on treated suspensions, with results presented in Figure 12.

As shown in Figure 12, the absorbance of the unsupported nano-TiO_2_ suspension was relatively low, at 0.372. With the increase in AC and Al_2_O_3_ support contents, the absorbance of the composite suspensions increased progressively, indicating effectively improved dispersion uniformity. Compared with the unsupported sample, the absorbance of AC-supported samples increased by 39.2%, 69.6%, 125.0%, and 134.7% at support contents of 20%, 30%, 40%, and 50%, respectively. For Al_2_O_3_-supported samples, the increases were 27.2%, 58.3%, 145.4%, and 159.9%, respectively. At support contents of 20% and 30%, the dispersion uniformity was only partially improved, suggesting insufficient support coverage for the modified nano-TiO_2_. When the support content reached 40%, the absorbance increased notably, indicating that the modified nano-TiO_2_ is fully supported on the surfaces of AC and Al_2_O_3_, effectively reducing agglomeration. At 50% support content, the rate of increase in absorbance diminished, suggesting that the support surfaces are saturated and optimal dispersion uniformity is achieved. Consistent with the fluorescence microscopy results, a support content of 40% for both AC and Al_2_O_3_ effectively reduced agglomeration and improved uniformity. Notably, the dispersion effect of Al_2_O_3_-supported nano-TiO_2_ is superior to that of the AC-supported samples. Therefore, Al_2_O_3_ with a support content of 40% is recommended as the optimal support for modified nano-TiO_2_.

### 3.3. Microstructural Characterization and Enhancement Mechanism

#### 3.3.1. Crystallographic Properties and Crystallite Size Effects

**Figure 13:**
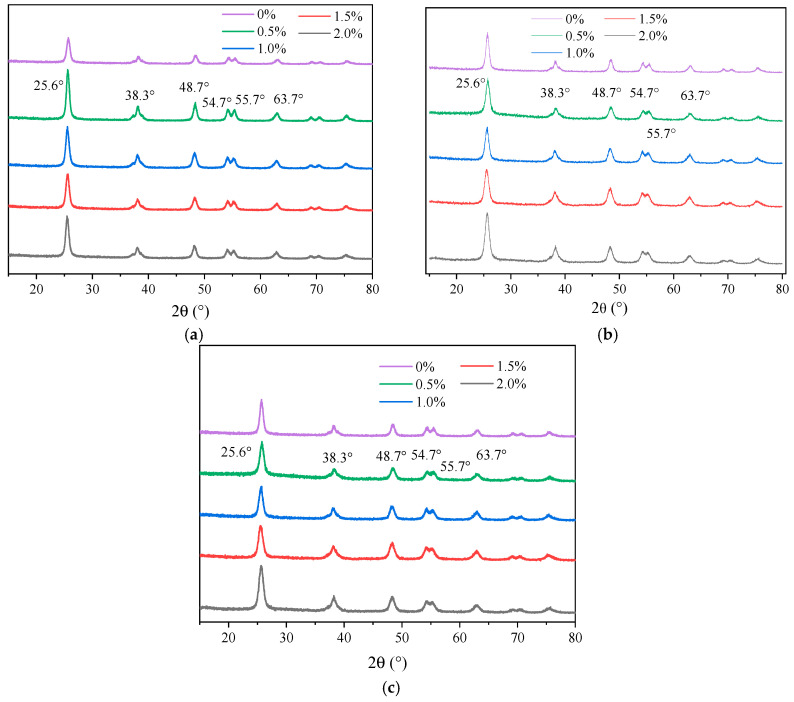
XRD patterns of metal ion-doped modified nano-TiO_2_. (**a**) Fe^3+^; (**b**) Ag^+^; (**c**) La^3+^.

The XRD patterns of nano-TiO_2_ doped with Fe^3+^, Ag^+^, and La^3+^ at various concentrations are presented in Figure 13.

As shown in Figure 13, the diffraction peaks of the unmodified nano-TiO_2_ were located at 2θ = 25.6°, 38.3°, 48.7°, 54.7°, 55.7°, and 63.7°, corresponding to the (101), (004), (200), (105), (211), and (204) crystal planes of anatase TiO_2_, respectively. This confirmed that the samples prepared via the sol–gel method using tetrabutyl titanate as the precursor consist primarily of the anatase phase [40]. Notably, no characteristic diffraction peaks corresponding to metal oxide impurities or secondary phases derived from Fe^3+^, Ag^+^, or La^3+^ were detected in any of the doped samples. However, the intensity and full width at half maximum of the diffraction peaks vary depending on the type and content of the doped metal ions. These changes suggested that during the doping process, metal ions substitute for Ti^4+^ ions within the nano-TiO_2_ lattice. Such substitution introduced lattice defects, inhibited crystal growth, and consequently altered the crystallite size of the materials [41].

**Figure 14:**
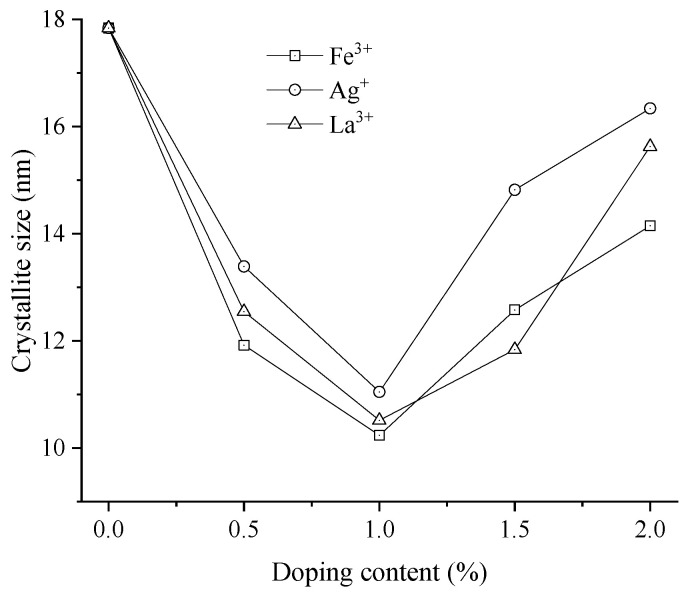
Crystallite sizes of metal ion-doped modified nano-TiO_2_.

Given that particle size is a critical parameter influencing the photocatalytic performance of the modified nano-TiO_2_ [42], rigorous characterization was conducted to quantify the effects of different metal ion doping modifications. The calculated crystallite sizes of the modified nano-TiO_2_ samples with different metal ion types and contents are presented in Figure 14.

As illustrated in Figure 14, the crystallite size of the modified nano-TiO_2_ varied obviously with metal ion content. The trend showed an initial decrease followed by an increase, reaching a minimum value at a doping content of 1.0%. This behavior aligned with recent studies on metal-doped TiO_2_, where low doping concentrations inhibit grain growth by restricting crystal nucleation and growth, while excessive doping leads to dopant agglomeration and subsequent crystallite coarsening [43]. At this optimal doping content, the crystallite sizes for Fe^3+^-, Ag^+^-, and La^3+^-doped nano-TiO_2_ were 10.24 nm, 11.05 nm, and 10.52 nm, respectively. These metal ions induced lattice contraction or distortion depending on their ionic radii relative to Ti^4+^. Specifically, Fe^3+^ possessed a radius similar to Ti^4+^, facilitating substitutional doping and lattice contraction, while larger ions (La^3+^ and Ag^+^) tended to occupy interstitial sites, causing lattice distortion. Smaller crystallite sizes enhanced the surface-to-volume ratio, effectively increasing the contact area with exhaust gases and promoting photocatalytic reaction kinetics [44]. Thus, the modulation of crystallite size via metal ion doping directly governed the photocatalytic performance and exhaust degradation efficiency.

**Figure 15:**
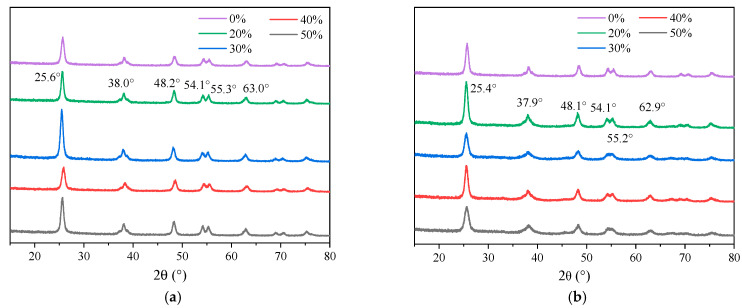
XRD patterns of nano-TiO_2_ supported by different supports. (**a**) AC; (**b**) Al_2_O_3_.

Moreover, the XRD patterns of the composite modified nano-TiO_2_, prepared by supporting 1.0% Fe^3+^-doped nano-TiO_2_ with AC and Al_2_O_3_, are shown in Figure 15.

As shown in Figure 15, the composite samples retained the characteristic diffraction peaks of anatase TiO_2_ at 2θ = 25.6°, 38.0°, 48.2°, 54.1°, 55.3°, and 63.0°. The preservation of this crystalline structure is crucial for maintaining high photocatalytic activity [45]. No characteristic peaks corresponding to crystalline carbon phases were detected, likely due to peak overlap or the amorphous nature of the activated carbon. Similarly, the absence of new peaks in the Al_2_O_3_-supported samples was attributed to the amorphous structure or low crystallinity of the aluminum oxide support. This suggested that the sol–gel preparation may induce interactions between Al and Ti species, potentially generating amorphous Ti-Al-O structures [46]. Furthermore, while the peak intensity varied with AC and Al_2_O_3_ content, the maintained peak positions indicated that the supports combine well with the modified nano-TiO_2_ without disrupting its crystalline framework. This structural compatibility provided a favorable basis for the improved dispersion of composite modified nano-TiO_2_ in the asphalt.

#### 3.3.2. Surface Chemical States and Hydroxyl Group Enrichment

**Figure 16:**
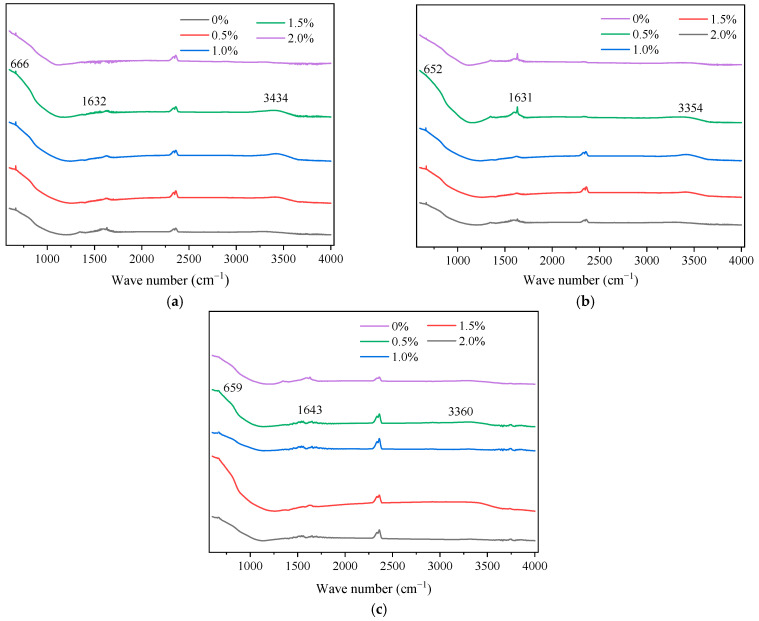
FTIR spectra of metal ion-doped modified nano-TiO_2_. (**a**) Fe^3+^; (**b**) Ag^+^; (**c**) La^3+^.

Complementing the crystallographic analysis, FTIR spectroscopy was employed to elucidate the surface chemical states and hydroxyl group enrichment of the metal ion-doped nano-TiO_2_ samples. The resulting infrared spectra are presented in Figure 16.

As depicted in Figure 16, the spectra of the metal ion-doped samples retained the fundamental structural features of unmodified nano-TiO_2_, with distinct absorption bands attributed to Ti-O-Ti stretching vibrations observed in the 500–800 cm^−1^ range. The absorption peak near 1632 cm^−1^ corresponded to the bending vibration of H-O-H bonds in absorbed water, while the broad band centered around 3473 cm^−1^ was assigned to the stretching vibration of O-H bonds in bound water molecules. The increased intensity of these peaks indicated that the modified nano-TiO_2_ has a greater affinity for atmospheric water adsorption, resulting in increased surface hydroxyl density. Since a higher concentration of surface -OH groups facilitates the generation of hydroxyl radicals under visible light irradiation, this enrichment directly contributes to enhanced photocatalytic activity and exhaust gas degradation efficiency [47]. Additionally, the absence of new characteristic peaks corroborated the XRD findings, confirming that metal ions are incorporated into the lattice without forming separate oxide phases. Regarding the influence of doping content, the absorption peak near 660 cm^−1^ exhibited a noticeable shift as the metal ion content increased, accompanied by a gradual weakening of the Ti-O bond intensity. Consistent with the lattice substitution mechanism identified in the XRD analysis, this indicated that Fe^3+^, Ag^+^, and La^3+^ ions substitute for Ti^4+^ within the lattice, disrupting lattice symmetry and reducing Ti-O absorption peak intensity [48]. Moreover, the integrated area of the absorption peaks initially increased and subsequently decreased with rising metal ion content.

**Figure 17:**
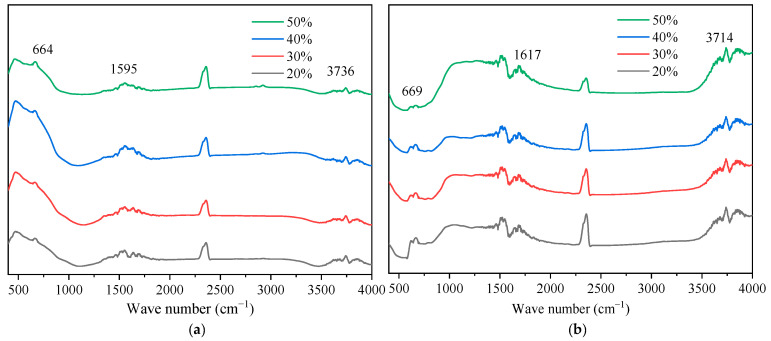
FTIR spectra of TiO_2_ supported by different supports. (**a**) AC; (**b**) Al_2_O_3_.

Furthermore, the FTIR spectra of the composite modified nano-TiO_2_ supported by AC and Al_2_O_3_ are presented in Figure 17.

As illustrated in Figure 17, the composite samples exhibited similar spectral profiles across different support contents. Characteristic absorption peaks were observed at 664.2, 1594.7, and 3736.1 cm^−1^ for the AC-supported samples, and at 668.6, 1617.4, and 3713.5 cm^−1^ for the Al_2_O_3_-supported samples. The intensity of these peaks varied with support content. Specifically, shifts in the Ti-O absorption band were attributed to interfacial interactions between the support materials and the nano-TiO_2_ lattice during sol–gel processing and calcination [49]. These interactions are critical for influencing the dispersion behavior of the composite samples. Additionally, the peaks near 1594.7 cm^−1^ and 3713.5 cm^−1^ (assigned to H-O-H bending and O-H stretching, respectively) showed increased intensity and area, indicating a further enrichment of surface hydroxyl groups induced by support modification. This enhancement introduces additional active sites and promotes hydroxyl formation, which effectively reduces agglomeration through improved surface compatibility. Consequently, this provides a chemical basis for the improved dispersion uniformity of the composite modified nano-TiO_2_ within the asphalt binder.

### 3.4. Performance Analysis of Photocatalytic Permeable Asphalt Mixture

To evaluate the exhaust degradation effect and pavement performance of the composite modified nano-TiO_2_, photocatalytic permeable asphalt mixtures were prepared by replacing mineral filler with composite modified nano-TiO_2_ at dosages of 30%, 40%, 50%, and 60%. Both exhaust degradation tests and pavement performance experiments were conducted on the prepared mixtures.

#### 3.4.1. Analysis of Exhaust Degradation Effect

**Figure 18:**
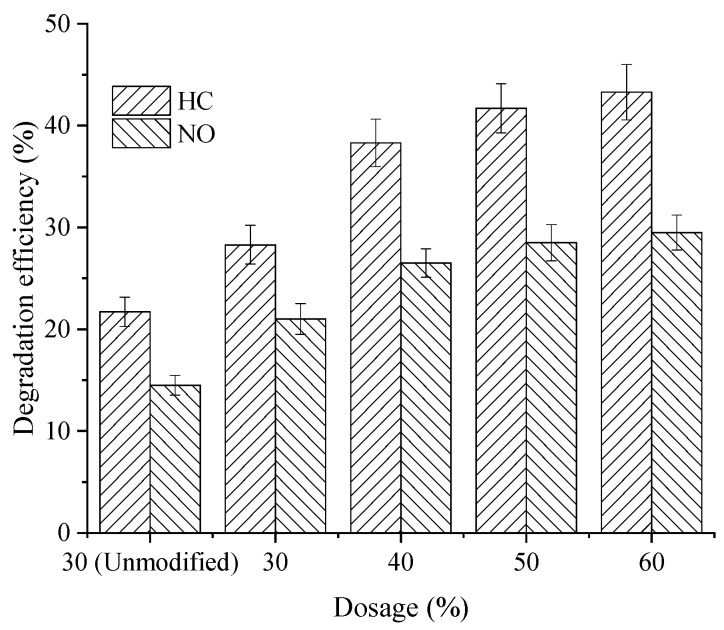
Exhaust degradation effect of composite modified nano-TiO_2_.

The variation in HC and NO degradation efficiency for the photocatalytic permeable asphalt mixtures with different composite modified nano-TiO_2_ contents is shown in Figure 18.

As shown in Figure 18, the incorporation of composite modified nano-TiO_2_ enhanced the HC and NO degradation efficiency of the permeable asphalt mixtures. Specifically, at composite modified nano-TiO_2_ dosages of 30%, 40%, 50%, and 60%, the HC degradation efficiencies reached 28.3%, 38.3%, 41.7%, and 43.3%, respectively, while the NO degradation efficiencies reached 21.0%, 26.5%, 28.5%, and 29.5%, respectively. The effect of dosage on degradation efficiency is consistent with the findings reported in previous study [3]. Compared with mixtures containing unmodified nano-TiO_2_, the HC and NO degradation efficiencies of the composite modified mixtures increased by 30.8% and 44.8%, respectively. These results indicated that Fe^3+^ doping and Al_2_O_3_ support treatment effectively improve the dispersion uniformity of nano-TiO_2_ in the asphalt mixture, thereby enhancing photocatalytic degradation performance for vehicle exhaust. However, the rate of improvement in degradation efficiency diminished when the composite modified nano-TiO_2_ dosage exceeded 50%. Therefore, a replacement ratio of 40–50% is recommended for preparing photocatalytic permeable asphalt mixtures to balance performance and material usage.

#### 3.4.2. Analysis of Pavement Performance

**Figure 19:**
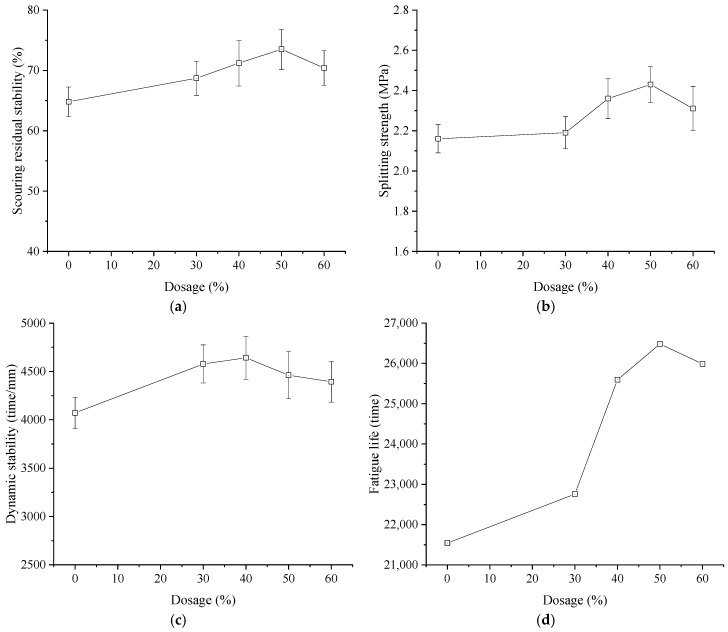
Pavement performance of photocatalytic permeable asphalt mixtures. (**a**) Scouring residual stability; (**b**) Splitting strength; (**c**) Dynamic stability; (**d**) Fatigue life.

The residual stability after water scouring, low-temperature splitting strength, dynamic stability, and fatigue life of photocatalytic permeable asphalt mixtures with different composite modified nano-TiO_2_ contents are presented in Figure 19.

As illustrated in Figure 19, after 3500 scouring cycles under hydrodynamic pressure at 30 °C [31], the residual stability of the photocatalytic permeable asphalt mixtures initially increased and subsequently decreased with increasing composite modified nano-TiO_2_ content. It should be noted that the water sensitivity was evaluated using the dynamic water scouring Marshall test. As there are no specified limits for this method, the control group without nano-TiO_2_ was used as the benchmark to evaluate the improvement. Compared with the control group, the scouring residual stability increased by 6.0%, 9.9%, 13.4%, and 8.6% at dosages of 30%, 40%, 50%, and 60%, respectively, reaching a maximum at 50% content. Similarly, the low-temperature splitting strength improved across all doping levels, indicating favorable cracking resistance. The splitting strength also peaked at a content of 50%, with an increase of 12.5% compared to the control group. Regarding high-temperature performance, the dynamic stability values for these mixtures substantially exceeded the standard specification limit of 3000 cycles/mm. The maximum dynamic stability of 4542 cycles/mm was achieved at 40% dosage, representing an 11.3% increase over the control group. Furthermore, the fatigue life of the mixtures improved to varying extents. Specifically, the fatigue life reached 26,483 cycles at 50% content, an increase of 22.9%. Moreover, the coefficient of variation for residual stability, splitting strength, dynamic stability and fatigue life is within 6.0%.

Evidently, the addition of composite modified nano-TiO_2_ enhanced the overall pavement performance of photocatalytic permeable asphalt mixtures to varying degrees. This improvement was attributed to the reduced crystallite size and enlarged specific surface area confirmed by XRD analysis. Fluorescence microscopy and UV-Vis analysis mentioned above also verified the uniform dispersion of the modifier within the asphalt binder. These factors induced microstructural reinforcement at the asphalt interface. The resulting transition layer strengthened the bonding between nanoparticles and asphalt, improving interfacial compatibility through the synergistic effects of chemical bonding and physical anchoring [50]. Additionally, the abundant surface hydroxyl groups evidenced by FTIR spectra retarded the oxidative hardening of the asphalt and form hydrogen or ester bonds with carboxylic groups in the asphalt binder. This enhanced the asphalt–aggregate interfacial bonding strength and inhibits the propagation of micro cracks along the aggregate interface [51]. Consequently, the low-temperature splitting strength, scouring resistance, high-temperature deformation resistance, and fatigue life were improved. It should be noted that while this study focused on initial performance, the gains in fatigue life and water scouring resistance suggested improved long-term durability potential. However, when the composite modified nano-TiO_2_ dosage was excessive, the dispersion quality decreased. This agglomeration disrupted the continuity of the asphalt mastic and created internal defects, thereby reducing the improvement effect of modified nano-TiO_2_ on pavement performance.

In terms of permeability performance, the permeability coefficient was validated during the mix design phase, confirming compliance with the specifications for permeable asphalt mixtures. As the composite modified nano-TiO_2_ was incorporated via volumetric replacement of mineral filler without altering the aggregate gradation, the permeability performance across modified groups remained consistent with the control group without nano-TiO_2_. Regarding the permeability–strength trade-off, conventional strengthening methods typically compromise permeability by reducing air void content. In contrast, the nano-modifier functions as a filler replacement while maintaining the aggregate skeleton, then the strength enhancements are derived from interfacial reinforcement rather than void reduction. This strategy effectively overcomes the performance conflict, delivering enhanced mechanical properties without compromising drainage capacity. Moreover, while contemporary photocatalytic research predominantly focuses on material synthesis [35], this study validated the engineering performance within the context of permeable asphalt mixtures. The dynamic stability and fatigue life exceeded typical values reported for conventional photocatalytic OGFC mixtures, confirming that the proposed composite strategy offers a superior balance of exhaust degradation and pavement performance.

## 4. Conclusions

The degradation efficiencies for HC and NO exhibit a trend of initial increase followed by a decrease with rising metal ion content. The maximum degradation efficiencies are achieved at doping concentrations of 1.0% for Fe^3+^, 1.5% for Ag^+^, and 1.0% for La^3+^. Notably, Fe^3+^-doped nano-TiO_2_ demonstrates superior exhaust degradation performance achieving efficiencies of 46.7% for HC and 33.5% for NO. Doping nano-TiO_2_ with 1.0% Fe^3+^ is recommended to maximize exhaust degradation efficacy.

The enhanced photocatalytic activity is attributed to metal ion doping-induced microstructural modifications. During modification, metal ions substitute for partial Ti^4+^ within the nano-TiO_2_ lattice, inducing lattice defects, inhibiting crystal growth, and reducing crystallite sizes. At the optimal doping content of 1.0%, crystallite size of Fe^3+^-doped sample minimizes to 10.24 nm, which increases the density of photo-absorption sites. Furthermore, the surface hydroxyl group density peaks at this content, facilitating more efficient photocatalytic reactions and directly influencing exhaust degradation performance.

Both AC and Al_2_O_3_ support improve the dispersion of modified nano-TiO_2_ within the asphalt binder. While AC-supported samples show superior dispersion at lower support contents of 20% and 30%, Al_2_O_3_-supported samples perform better at a 40% content. At this optimal support content, Al_2_O_3_ supports yield a smaller range of area ratios and higher suspension absorbance, effectively mitigating agglomeration and enhancing uniformity. Al_2_O_3_ with a 40% support content is recommended as the optimal support to ensure uniform dispersion of modified nano-TiO_2_ in asphalt.

The improved dispersion properties are governed by the structural compatibility between the support and nano-TiO_2_. The absence of new diffraction peaks in the AC- and Al_2_O_3_-supported composites confirms that the supports load effectively without disrupting the TiO_2_ crystalline structure. Moreover, the supports introduce additional surface active sites and promote hydroxyl group formation through interfacial interactions. These surface modifications reduce the agglomeration tendency of the modified nano-TiO_2_, thereby improving its dispersion and long-term stability within the asphalt binder.

Balancing exhaust degradation performance with pavement engineering properties, the optimal dosage of composite modified nano-TiO_2_ is determined to be 40–50% of the mineral filler mass. Within this range, the composite modifier enhances the pavement performance of photocatalytic permeable asphalt mixtures via physical filling and micro-structural optimization. Specifically, at a 50% replacement ratio, the dynamic stability reaches 4462 cycles/mm and the fatigue life improves to 26,483 cycles, demonstrating excellent engineering applicability.

Despite the promising results, certain limitations are acknowledged in the scope of this study. Permeability performance was inferred from the constant aggregate skeleton established during mix design rather than directly tested for each modifier dosage. Additionally, while statistical variability was assessed via coefficient of variation and error bars, and fatigue tests suggest durability, long-term aging performance was not included. Future work will focus on direct permeability validation under clogging conditions and long-term aging evaluations to further confirm service life.

## Figures and Tables

**Table 1:** Raw materials of the composite modified nano-TiO_2_.

Raw Materials	Molecular Formula	Specification	Manufacturer
Butyl titanate	C_16_H_36_O_4_Ti	AR	Shanghai Zhanyun Chemical Co., Ltd. (Shanghai, China)
Iron (III) nitrate nonahydrate	Fe(_NO3_)_3_·9H_2_O	AR	Shanghai Zhanyun Chemical Co., Ltd.
Lanthanum nitrate hexahydrate	La(_NO3_)_3_·6H_2_O	AR	Shanghai Zhanyun Chemical Co., Ltd.
Anhydrous ethanol	C_2_H_6_O	AR	Bengbu Chemical Reagent Factory (Bengbu, China)
Glacial acetic acid	CH_3_COOH	AR	Tianjin Damao Chemical Reagent Co., Ltd. (Tianjin, China)
Distilled water	—	—	—
Hydrochloric acid	HCl	AR	Sinopharm Chemical Reagent Co., Ltd. (Beijing, China)
Silver nitrate	AgNO_3_	AR	Sinopharm Chemical Reagent Co., Ltd.
Nitric acid	HNO_3_	AR	Sinopharm Chemical Reagent Co., Ltd.

## Data Availability

The original contributions presented in this study are included in the article. Further inquiries can be directed to the corresponding author.
